# Construction of an activatable ratiometric fluorescent nanoprobe for HClO detection and imaging in acute kidney injury models

**DOI:** 10.1039/d6ra04230a

**Published:** 2026-07-08

**Authors:** Tianhui Wu, Zhihui Li, Liang Zhang, Zhijuan Kang, Mai Xun, Hanyao Hua, Wei Zhang

**Affiliations:** a Department of Nephrology, Rheumatology and Immunology, The Affiliated Children's Hospital of Xiangya School of Medicine, Central South University (Hunan Children's Hospital) Changsha Hunan 410007 China 18374970668@163.com Lizh0731@aliyun.com; b College of Food Science and Engineering, Central South University of Forestry and Technology Changsha Hunan 410004 China

## Abstract

Acute kidney injury (AKI) is a prevalent and life-threatening clinical condition associated with substantial morbidity and mortality. Early diagnosis and prompt therapeutic intervention are therefore critical to improving patient prognosis. A hallmark pathophysiological feature of AKI—regardless of etiology, including ischemia/reperfusion injury, nephrotoxic drug exposure, or sepsis—is the burst-like overproduction of hypochlorous acid (HClO). Consequently, dynamic and quantitative monitoring of renal HClO levels not only advances our mechanistic understanding of AKI progression but also holds significant promise for early disease warning and real-time assessment of therapeutic efficacy. Herein, we developed an HClO-activatable ratiometric nanofluoroprobe, designated CHI-CBO, by covalently conjugating a small-molecule HClO-responsive fluorophore to glycol chitosan—a biocompatible and biodegradable natural polysaccharide. CHI-CBO exhibits a robust ratiometric fluorescence response to HClO and demonstrates prolonged renal retention, enabling sustained and high-fidelity imaging. Notably, CHI-CBO achieved high-performance dynamic ratiometric fluorescence imaging of endogenous HClO in living RAW264.7 macrophages and zebrafish embryos. Moreover, using CHI-CBO as an imaging agent, we successfully performed real-time, noninvasive ratiometric fluorescence imaging of HClO accumulation in the kidneys of a murine AKI model—thereby underscoring its translational potential for early AKI detection and monitoring.

## Abstract 2

An activatable ratiometric fluorescence imaging of HClO accumulation in the kidneys of a murine AKI model—thereby underscoring its translational potential for early AKI detection and monitoring.

## Graphical abstract

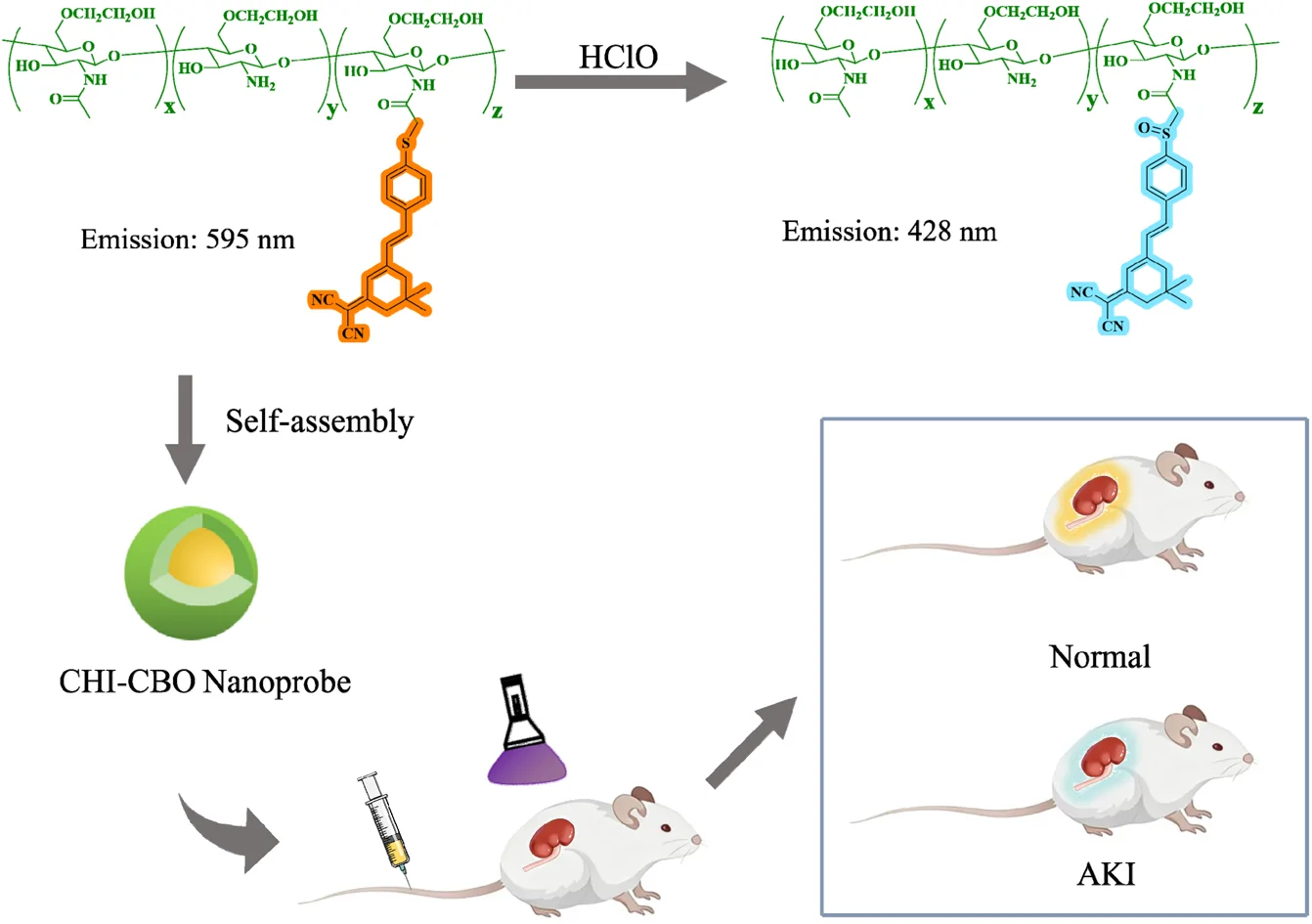

## 1 Introduction

The pathological characteristics of acute kidney injury (AKI) are a rapid decline in glomerular filtration rate and a sharp accumulation of nitrogenous waste, affecting 10–15% of hospitalized patients and strongly associated with chronic kidney disease, end-stage renal disease, and mortality risk.^1–3^ Current clinical diagnosis mainly relies on serum creatinine and changes in urine output^4–6^ These indicators have low sensitivity and significant lag, often showing abnormalities only several hours to days after functional damage has occurred, missing the ‘golden window’ for early intervention. Therefore, developing biomarkers and imaging technologies capable of detecting early cellular and molecular injuries has become an urgent need in the field of AKI research.

Oxidative stress plays a central role in the complex pathological network of acute kidney injury.^7–9^ The kidney, as a high oxygen-consuming organ rich in mitochondria, is extremely sensitive to redox imbalance.^10^ Under pathological stimuli such as ischemia or exposure to toxins, excessive levels of reactive oxygen species (ROS)—including the superoxide anion (O_2_˙^−^), hydrogen peroxide (H_2_O_2_), and hypochlorous acid (HOCl)—are generated primarily by enzymatic sources such as the NADPH oxidase (Nox) family and the mitochondrial electron transport chain, far exceeding the clearance capacity of endogenous antioxidant systems (such as superoxide dismutase and glutathione).^11,12^

This oxidative stress-mediated damage compromises cellular membrane integrity, impairs enzymatic activity, and disrupts mitochondrial function, ultimately triggering apoptosis, necrosis, and an inflammatory cascade response in renal tubular epithelial cells, ultimately promoting the occurrence and progression of AKI.^13–15^ A large body of evidence suggests that ROS is an early driver and key biomarker of AKI.^16,17^ Among these ROS, hypochlorous acid and its conjugate base (hypochlorite ion), are common strong oxidants found in our daily life and within living organisms. Studies have shown that under physiological conditions, about half of HClO dissociates into ClO^−^, and excessive production of HClO is associated with various human diseases, such as cardiovascular diseases,^18^ neurodegenerative disorders,^19^ arthritis,^20^ and even cancer.^21^ In addition, HClO may cause lysosomal rupture, thereby affecting cell apoptosis.^22^ Therefore, visualizing the dynamic changes of HClO within the kidney is equivalent to directly observing the initiation and progression of AKI, which has unparalleled scientific value and clinical potential.^23–25^

Prior to the development of nanoprobes, small-molecule fluorescent probes were widely used for hypochlorous acid (HClO) detection.^26–30^ However, these probes have inherent limitations: (1) limited tissue penetration: their emission/excitation wavelengths mostly fall in the ultraviolet-visible region, which suffers from significant tissue absorption and scattering, making them difficult to use for *in vivo* imaging of deep organs (such as the kidneys); (2) insufficient specificity, leading to false positives; (3) lack of targeting: they distribute systemically, with low accumulation in the kidneys and poor signal-to-noise ratio; (4) suboptimal pharmacokinetics: unable to achieve long-term monitoring.

The advent of nanotechnology has established a transformative platform for significantly enhancing the performance of fluorescent probes. Fluorescent nanoprobes are typically constructed using inorganic nanoparticles (such as gold nanoclusters and quantum dots), organic polymer nanoparticles, silica nanoparticles, or self-assembled nanostructures as carriers, by loading or covalently attaching analyte-responsive fluorophores. Their advantages are reflected in ref. 31–37: (1) enhanced penetration and signal-to-noise ratio: by designing probes that emit wavelengths in the relatively long wavelength, tissue autofluorescence and light scattering can be significantly reduced, enabling deeper and clearer *in vivo* imaging. (2) Excellent targeting capability: by modifying the surface with kidney-targeting ligands (such as low-molecular-weight proteins or specific peptides), probes can achieve specific accumulation in the kidneys, improving detection sensitivity. (3) Diverse response mechanisms: various sensing modes, such as ratiometric, reversible, and activatable, can be integrated to enable quantitative and dynamic monitoring of analyte concentration changes, rather than simple “on-off” signaling. (4) Potential for integrated diagnosis and therapy: nanocarriers can simultaneously carry analyte-responsive fluorescent reporting units and antioxidant therapeutic drugs (or possess intrinsic enzyme-like activity), achieving targeted therapy while diagnosing, *i.e.*, “theranostics”.

**Scheme 1:**
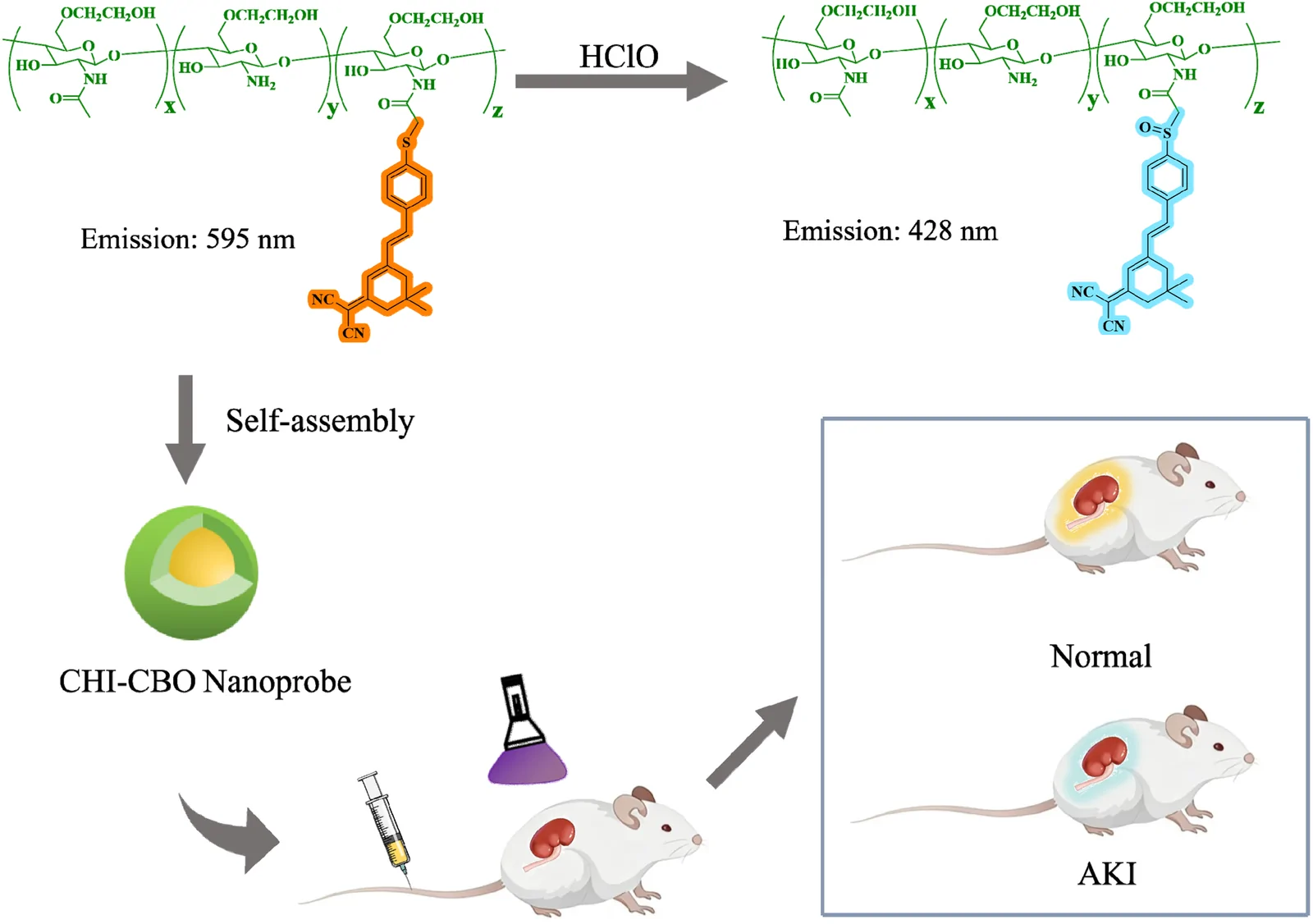
Schematic illustration of the working principle of HClO-activated fluorescent nanoprobe CHI-CBO for AKI detection *via in vivo* fluorescence imaging.

To address these challenges, we developed a HClO-activated small-molecule fluorescent probe (a dicyanomethylene isophorone derivative), grafted onto chitosan to design a novel HClO-activated ratiometric fluorescent nanoprobe, named CHI-CBO. The CHI-CBO exhibits a large Stokes shift of 146 nm to avoid excitation-induced scattering effects; maximum emission in the far-red region (595 nm) for deeper tissue penetration; and has two distinct emission bands (486 nm and 586 nm), with a Δ*λ* of 100 nm to ensure high accuracy in measuring the emission ratio. Ultimately, CHI-CBO was successfully applied for fluorescent imaging characterization of HClO changes in Raw264.7 cells, zebrafish, and AKI mouse models (Scheme 1).

## 2 Experimental

### 2.1 Materials and instruments

All chemical raw materials were purchased from Energy-Chemical, J&K Scientific, and Sigma-Aldrich and were used without further purification unless otherwise stated. Detailed procedures for optical detection, testing solution preparation, cell/zebrafish culture, AKI mouse model construction, and fluorescent imaging procedure are provided in the SI.

### 2.2 Fluorescent imaging of HClO in RAW264.7 cells and zebrafish by microscopy

To investigate the imaging performance of nanoprobes CHI-CBO for hypochlorous acid at different hypochlorous acid concentrations, RAW264.7 cells were seeded at a density of 1.0 × 10^4^ cells per well in 96-well plates and incubated at 37 °C for 24 h in 200 µL of culture medium. The culture medium was then replaced with fresh culture medium containing 0, 10, and 20 µM of hypochlorous acid, respectively, and 20 µg mL^−1^ of nanoprobes CHI-CBO was added, and after 4 h of incubation, the cells were washed three times with Dulbecco's phosphate-buffered saline (DPBS) for imaging. In the case of the lipopolysaccharide (LPS) stimulation, the cells were pre-treated with 1 µg mL^−1^ of LPS for 12 h, then washed with DPBS. Then, 20 µg mL^−1^ of nano-probes CHI-CBO was added, and after 4 h of incubation, the culture medium was replaced with DPBS for the imaging experiment. Image pixel fluorescence intensity was analyzed using Image-Pro Plus software. Each analysis was repeated three times, and the standard deviation was calculated as the error bar, *λ*_ex_ = 380 nm, fluorescent emission reception range blue channel (400–450 nm) and the red channel (600–630 nm), scale bar 50 µm.

Zebrafish housing and maintenance: adult zebrafish (*Danio rerio*) were obtained from the Model Animal Research Center of Central South University and housed under standardized conditions (28.5 °C; 14 h light/10 h dark cycle). Embryos were collected and reared in E3 medium (5 mM NaCl, 0.17 mM KCl, 0.33 mM CaCl_2_, 0.33 mM MgSO_4_). All experiments were conducted using 3 days-old larvae. Imaging procedure: for fluorescence imaging, 3 days-old larvae were incubated with 20 µg mL^−1^ CHI-CBO in E3 medium for 30 min at 28.5 °C. Following three washes with DPBS, larvae were exposed to 0, 10, or 20 µM HOCl for an additional 30 min and subsequently washed again. For LPS-induced inflammation modeling, larvae were pre-treated with 1 µg mL^−1^ LPS for 12 h, washed thoroughly, and then incubated with CHI-CBO for 4 h. Fluorescence imaging was performed on an inverted fluorescence microscope (Nikon Ti2) equipped with a 380 nm excitation source. Emission signals were collected simultaneously in the blue channel (400–450 nm) and the red channel (600–630 nm). Image pixel fluorescence intensity was analyzed using Image-Pro Plus software. Each analysis was repeated three times, and the standard deviation was calculated for the error bar, *λ*_ex_ = 380 nm, fluorescent emission reception range blue channel (400–450 nm) and the red channel (600–630 nm), scale bar 100 µm.

### 2.3 Fluorescence imaging test of AKI model

To elucidate the distribution of the nanoprobe *in vivo*, the nanoprobe CHI-CBO (100 µL, 20 mg mL^−1^ dissolved in water, the same below) was administered intravenously through the tail vein, and the organs (kidney, heart, liver, lung, and spleen) of the mice were imaged 30 min later (*λ*_ex_/*λ*_em_ = 560/600 nm, the same below). To induce acute injury (AKI), mice received an intraperitoneal injection of cisplatin (40 mg kg^−1^, dissolved in 300 µL saline) 48 h prior to imaging.^38^ Subsequently, the CHI-CBO was intravenously injected into the mice in this model group, and the imaging was performed for 30 min to compare the fluorescence signal intensity in the kidney area. The mice were then euthanized, and the organs were separated and imaged to determine the fluorescence distribution in each organ. Finally, kidneys were harvested and sectioned for imaging, and the differences in fluorescence distribution were compared, *λ*_ex_ = 380 nm, fluorescent emission reception range blue channel (400–450 nm) and the red channel (600–630 nm), scale bar 150 µm.

## 3 Results and discussion

### 3.1 Design and synthesis of nanoprobe

**Fig. 1:**
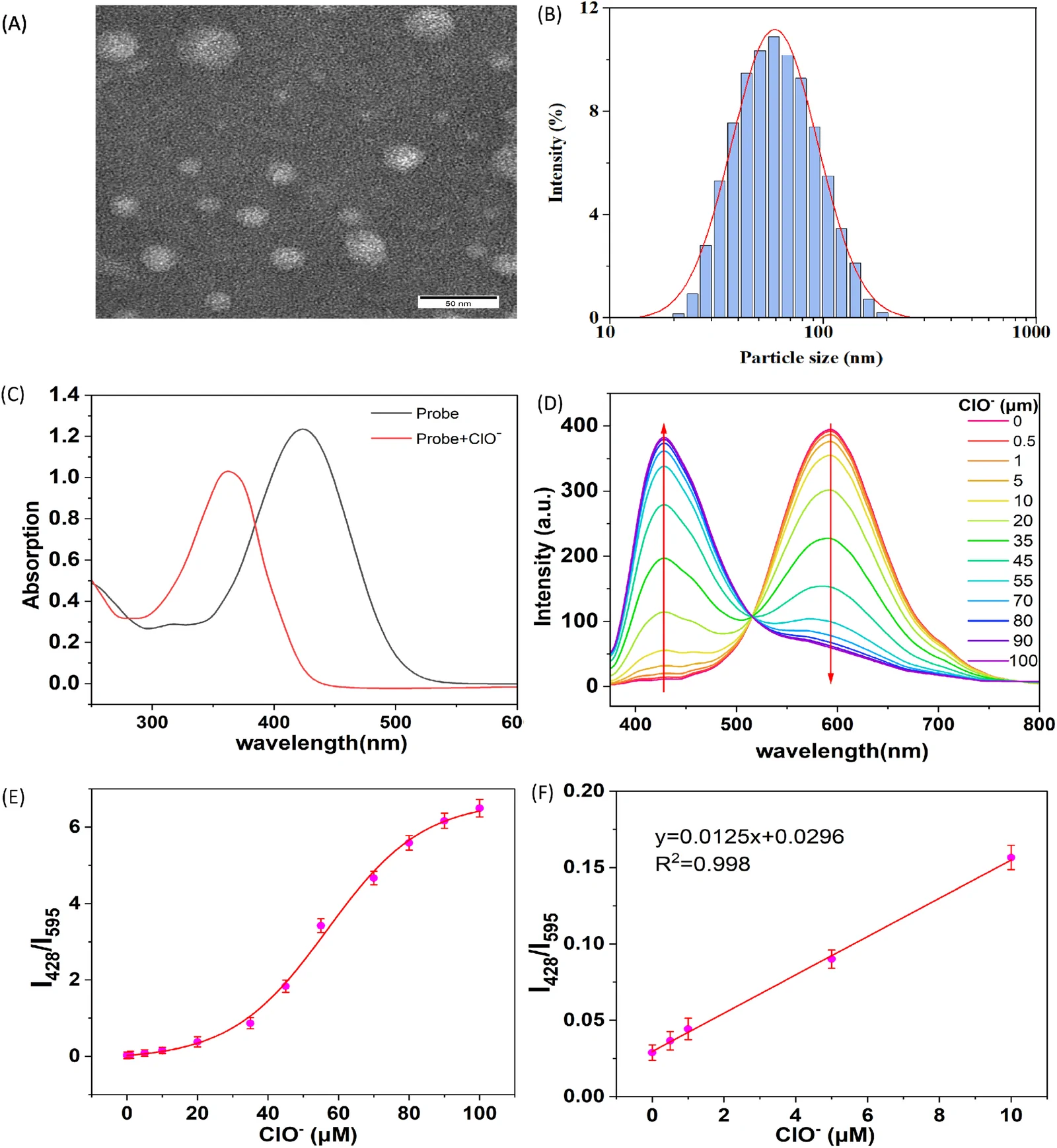
(A) TEM image of CHI-CBO showing self-assembled nanoparticles. The observed size distribution (5–60 nm) is characteristic of polymer-based micellar systems prepared by dialysis—a method known to yield polydisperse populations due to the kinetic nature of the self-assembly process. The average hydrodynamic diameter, determined by DLS (Fig. 1B), was 35.2 nm (PDI = 0.21), confirming the formation of nanoparticles with dimensions suitable for biological applications. (B) Particle size distribution curve of CHI-CBO. (C) Absorption. (D) Fluorescence spectra of CHI-CBO (20 µg mL^−1^). (E) Curves of the ratio of fluorescence intensity (*I*_428_/*I*_595_) of CHI-CBO (20 µg mL^−1^) reacted with (0–100) µM ClO^−^. (F) Linear correlation of *I*_428_/*I*_595_*vs.* ClO^−^ (0–10 µM). *λ*_ex_ = 375 nm, slit width: *d*_ex_ = 5 nm, *d*_em_ = 5 nm.

In order to design a highly selective HClO fluorescent probe, it is crucial to identify the specific reaction between the recognition group and HClO. Thiols (R_1_–S–R_2_) are readily oxidized to sulfoxides. Accordingly, we incorporated a thiol moiety as the recognition unit for HClO, which does not easily interfered by other bioactive molecules. Moreover, ideal probes for quantitative HClO in cells are required to have a ratiometric fluorescence readout and membraneability. Leveraging the intramolecular charge transfer (ICT) strategy, a highly efficient sulfur atom-dicyano-based “push–pull” system—constructed on a dicyanovinyl ketone scaffold—was employed to develop a ratiometric fluorescent probe capable of circumventing interference arising from variations in fluorophore concentration, instrumental fluctuations, and environmental factors. First, the small-molecule fluorescent probe 3 was synthesized *via* a straightforward substitution followed by a condensation reaction (Scheme S1). The structure of the probe was fully characterized by ^1^H NMR, and ^13^C NMR spectra (Fig. S3–S6), and we also verified the response mechanism of the small-molecule probe 3 with ClO^−^ using mass spectrometry (Fig. S1). Next, considering the water solubility, biocompatibility, and the long-term retention of the probe molecule in the kidney, we then grafted the designed small molecule probe 3 onto glycol chitosan (GC) to obtain a nanoprobe CHI-CBO, and the successful construction and detection performance of the nanoparticles were confirmed by Transmission Electron Microscope (TEM) (Fig. 1A), Dynamic Light Scattering (DLS) (Fig. 1B), and spectral responses (Fig. 1C and D).

### 3.2 Optical properties of nanoprobe CHI-CBO and its response to HClO

Since the application of a pure buffer system in biological detection is ideal, in this work, we chose PBS as the testing system to study the response of CHI-CBO toward HClO. The spectral characteristics of CHI-CBO were investigated under physiological conditions (PBS, 10 mM, pH 7.4). Firstly, the optical properties of the CHI-CBO were investigated in PBS buffer (10 mM, pH = 7.4). When the CHI-CBO reacted with 0 µM and 100 µM ClO^−^, the maximum absorption peaks of CHI-CBO and CHI-CBO + ClO^−^ were 375 nm and 455 nm, respectively (Fig. 1C). Upon reaction with ClO^−^, the absorption spectrum of CHI-CBO exhibited an 80 nm red shift. When excited at 375 nm, the CHI-CBO fluorescence at 595 was significantly weakened, and a new emission peak appeared at 428 nm, the intensity of which was significantly enhanced with the increase of ClO^−^ (Fig. 1D). More importantly, the concentration of ClO^−^ changed from (0–100) µM, the CHI-CBO's sulfide was oxidized into sulfoxide by sodium hypochlorite (Fig. S1), the CHI-CBO exhibited a ratiometric response, characterized by attenuation of the intramolecular charge-transfer (ICT) effect upon oxidation of the thioether to the electron-withdrawing sulfoxide (Fig. 1D), and can also be known from the structure of the nanoprobe CHI-CBO that the classic “A (dicyano)-π-D (sulfide)” structure becomes “A (dicyano)-π-A (sulfoxide)” structure. In addition, it can be seen from Fig. 1E that the value of the ratio response of the probe to hypochlorous acid significantly increased by 6-fold, and the concentration of ClO^−^ had a good linear response from 0 µM to 10 µM (Fig. 1F), and the LOD of CHI-CBO for ClO^−^ detection by 3*σ*/slope was estimated as 64.4 nM, its regression equation was *y* = 0.0125*x* + 0.0296 (*R*^2^ = 0.998), which exhibited that our nanoprobe CHI-CBO can quantitatively detect ClO^−^ under physiological conditions (Fig. 1D).

### 3.3 Selectivity of nanoprobe CHI-CBO

**Fig. 2:**
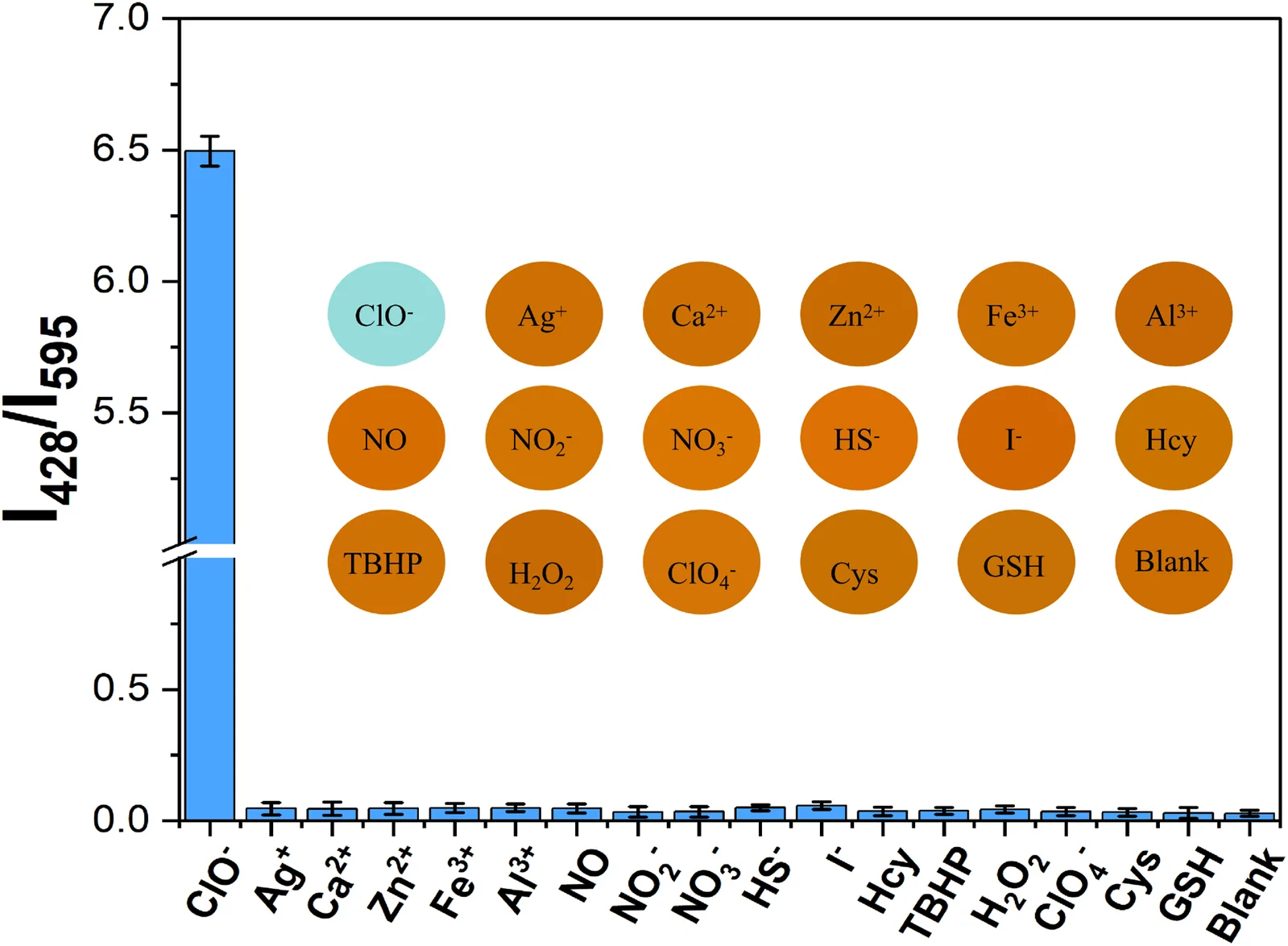
Fluorescence spectra of CHI-CBO (20 µg mL^−1^) in the presence of different anions in PBS: ClO^−^, Ag^+^, Ca^2+^, Zn^2+^, Fe^3+^, Al^3+^, NO, NO_2_^−^, NO_3_^−^, HS^−^, I^−^, Hcy, TBHP, H_2_O_2_, ClO_4_^−^, Cys, Blank, respectively. Insets display the corresponding fluorescence images acquired under 365 nm UV illumination following incubation with various analytes. *λ*_ex_ = 375 nm, slit width: *λ*_ex_ = 5 nm, *λ*_em_ = 5 nm, respectively.

To evaluate the specific fluorescence response of CHI-CBO toward hypochlorite (ClO^−^), we systematically assessed its selectivity against a panel of biologically relevant species. In one set of experiments, CHI-CBO (20 µg mL^−1^) was incubated with a mixture containing 100 µM ClO^−^ and 100 µM each of potential interfering species, namely, Ag^+^, Ca^2+^, Zn^2+^, Fe^3+^, Al^3+^, NO, NO_2_^−^, NO_3_^−^, HS^−^, I^−^, homocysteine (Hcy), *tert*-butyl hydroperoxide (TBHP), H_2_O_2_, ClO_4_^−^, and cysteine (Cys). As illustrated in Fig. 2 (blue bars), the presence of these interferents exerted no significant effect on the *I*_428_/*I*_595_ fluorescence intensity ratio elicited by ClO^−^, confirming the probe's exceptional selectivity. In a complementary experiment designed to visually demonstrate selectivity, a silicone film was immersed in a CHI-CBO solution and subsequently air-dried. The dried film was then cut into uniform pieces, each of which was individually immersed in an aqueous solution containing a single analyte (100 µM for all species, except 100 µM ClO^−^). Fluorescence images captured under 365 nm UV irradiation are presented as insets in Fig. 2. Notably, only the film piece exposed to ClO^−^ exhibited a distinct and pronounced fluorescence color shift from brick yellow to bright blue–green. A blank control was prepared by immersing a separate film piece in phosphate-buffered saline (PBS) devoid of any analyte. It can be seen from the image that only ClO^−^ can induce a change in the fluorescence color, from bright brick yellow to bright blue–green fluorescence emission. Therefore, it can be speculated that the CHI-CBO has the potential to respond to the fluctuation of HClO in a complex biological system.

### 3.4 Cytotoxicity testing and fluorescence imaging of nanoprobe CHI-CBO in living cells

**Fig. 3:**
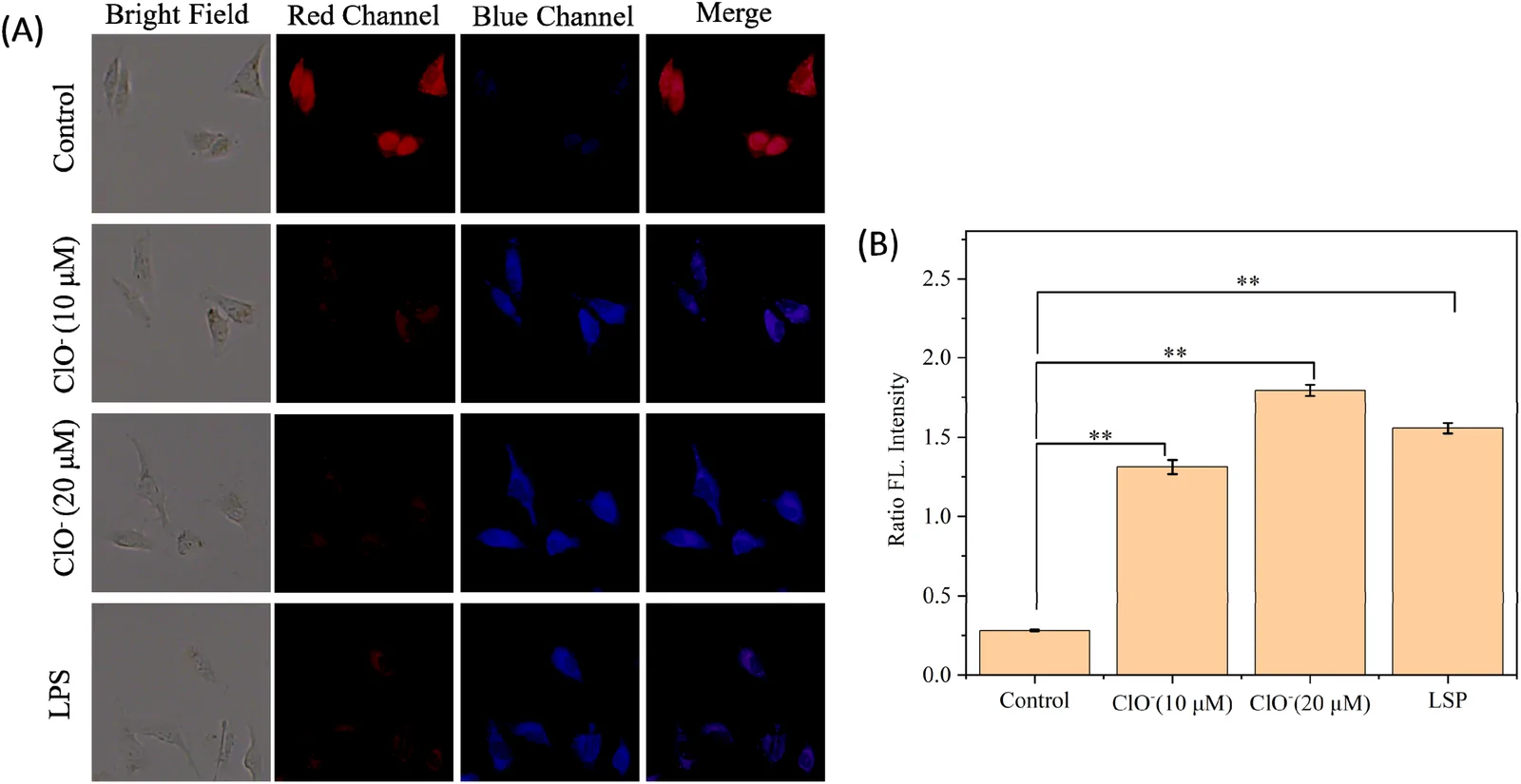
Living RAW264.7 cells fluorescent imaging. (A) The first line fluorescent images of RAW264.7 cells (control group), CHI-CBO incubated with the cells for 30 min; the second and third line fluorescent images of RAW264.7 cells are experimental groups, after 30 min of incubation of CHI-CBO with the cells, followed by 30 min of incubated with 10 µM and 20 µM ClO^−^; the fourth line fluorescent image of RAW264.7 cells is the cells stimulated by LPS, then incubated with CHI-CBO for 30 min. (B) Quantified ratio of relative fluorescence intensity (*I*_Blue_/*I*_Red_). Error bars: mean ± SD, *n* = 3. Scale bar: 50 µm, *λ*_ex_ = 380 nm. **: *p* < 0.05.

Motivated by the outstanding optical performance and selectivity of CHI-CBO, we proceeded to evaluate its efficacy in living systems. First, we used MTT staining experiments to study the cytotoxicity of the CHI-CBO, using RAW264.7 cells as a model. As shown in Fig. S2, it was obvious that the probe at (0–50) mg mL^−1^ had no obvious cytotoxicity on RAW264.7 cell lines after 24 h of incubation, and its excellent biocompatibility indicated that the probe could be further applied to *in vivo* or *in vitro* imaging analysis. In addition, we utilized the probe to evaluate the changes of HClO in RAW264.7 cells. First, the living RAW264.7 cells were incubated with the probe for 30 min. As a result, only the red channel showed a bright fluorescence signal, while the blue channel was weak (Fig. 3A: first line). Second, the cells were incubated with the probe for 30 min, and then the culture medium was replaced with a PBS buffer solution containing sodium hypochlorite (10 µM and 20 µM), and then incubated for another 30 min. The red channel decreased with the increase of HClO concentration, while the blue channel increased with the increase of HClO concentration, indicating that the fluorescence in cells depended on the change of HClO concentration (Fig. 3A: second & third line). Finally, to study the detection of endogenous HClO by the nanoprobe, the cells were stimulated with LPS after incubation with the probe, and the fluorescence signal in the red channel was weakened, while the fluorescence signal in the blue channel was enhanced (Fig. 3A: fourth line). Moreover, it was also known from the significance analysis diagram that the probe could indeed detect the changes of HClO in cells (Fig. 3B). Therefore, all these results were completely consistent with the detection response of the above probe in PBS buffer, which indicated that the probe was suitable for detecting the changes of HClO concentration in living RAW264 cells.

### 3.5 Fluorescence imaging of nanoprobe CHI-CBO in living zebrafish

**Fig. 4:**
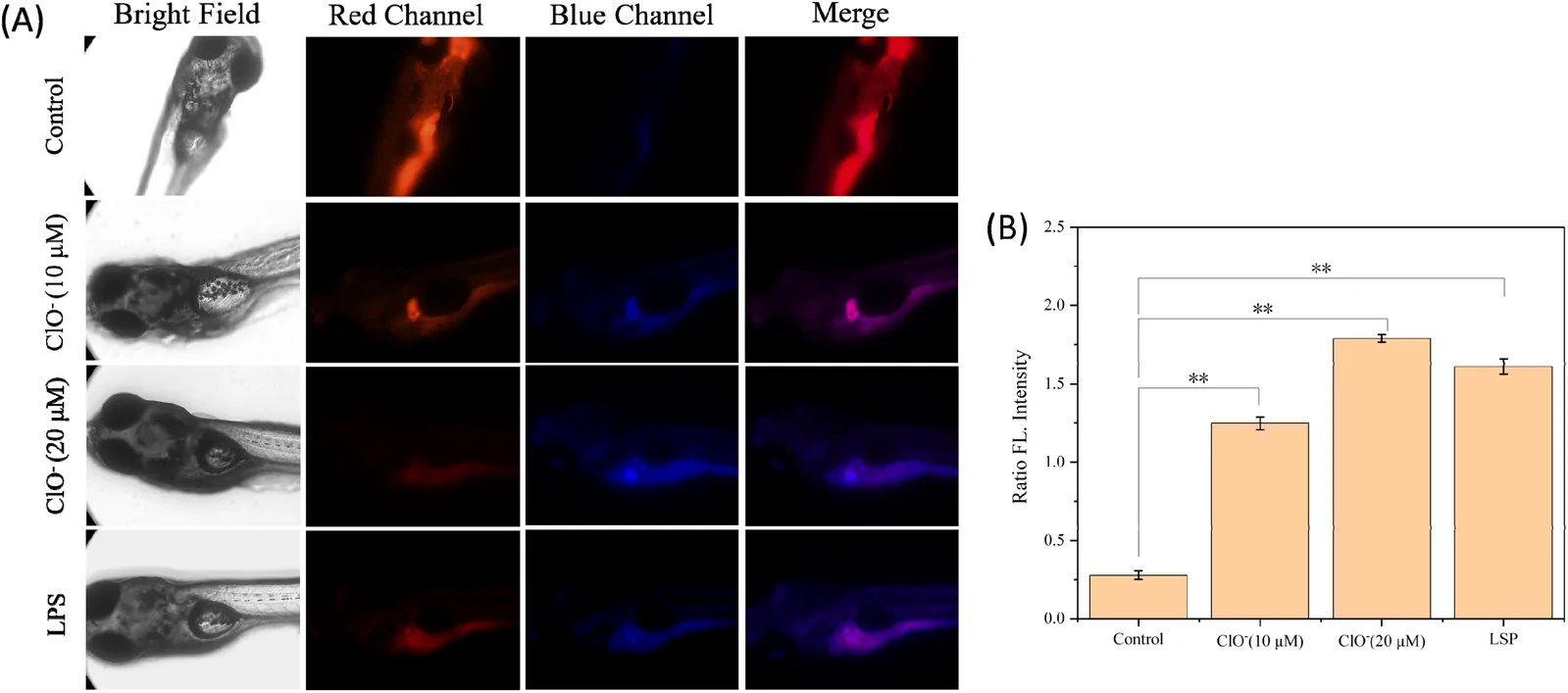
Zebrafish fluorescence imaging. (A) The first line fluorescent images of zebrafish is the control group, the probe incubated with the cells for 30 min; the second and third lines fluorescent images of zebrafish are experimental groups, after 30 min of incubation of the probe with the cells, followed by 30 min of incubated with 10 µM and 20 µM sodium hypochlorite; the fourth line fluorescent image of zebrafish is the cells stimulated by LPS, then incubated with CHI-CBO for 30 min. (B) Quantified ratio of relative fluorescence intensity (*I*_Blue_/*I*_Red_). Error bars: mean ± SD, *n* = 3. Scale bar: 100 µm. *λ*_ex_ = 380 nm. **: *p* < 0.05.

Encouraged by the satisfactory fluorescent imaging performance of the CHI-CBO in detecting HClO in RAW264.7 cells, we further studied the potential application analysis of the probe in tracking HClO changes in zebrafish. As shown in the first row of Fig. 4A, a zebrafish treated with CHI-CBO alone exhibited strong red fluorescence emission in the red channel under normal physiological conditions, while the blue channel was quite weak, indicating that the probe could penetrate the zebrafish (Fig. 4A: first line). In contrast, two groups of zebrafish incubated with CHI-CBO and then incubated with HClO at different concentrations (10 µM and 20 µM) showed strong blue emission in the blue channel and weak red emission in the red channel (Fig. 4A: second line & third line). Thus, the change of fluorescence intensity in zebrafish relied on the concentration change of HClO, a significant enhancement of the blue emission signal in the blue channel, and a significant decrease of the red emission signal in the red channel. In addition, with the stimulation of zebrafish with LPS, the same result was obtained: the red signal significantly weakened in the red channel, and the blue emission intensity was significantly enhanced in the blue channel (Fig. 4: fourth line), and the significance analysis of Fig. 4B also indicated that the probe could detect the change of HClO in zebrafish. Therefore, the experimental results, like the results of cell imaging, showed that the CHI-CBO can be used as a dual-channel fluorescent imaging for detecting the change of HClO *in vivo*.

### 3.6 Fluorescence imaging of nanoprobe CHI-CBO in AKI mouse

**Fig. 5:**
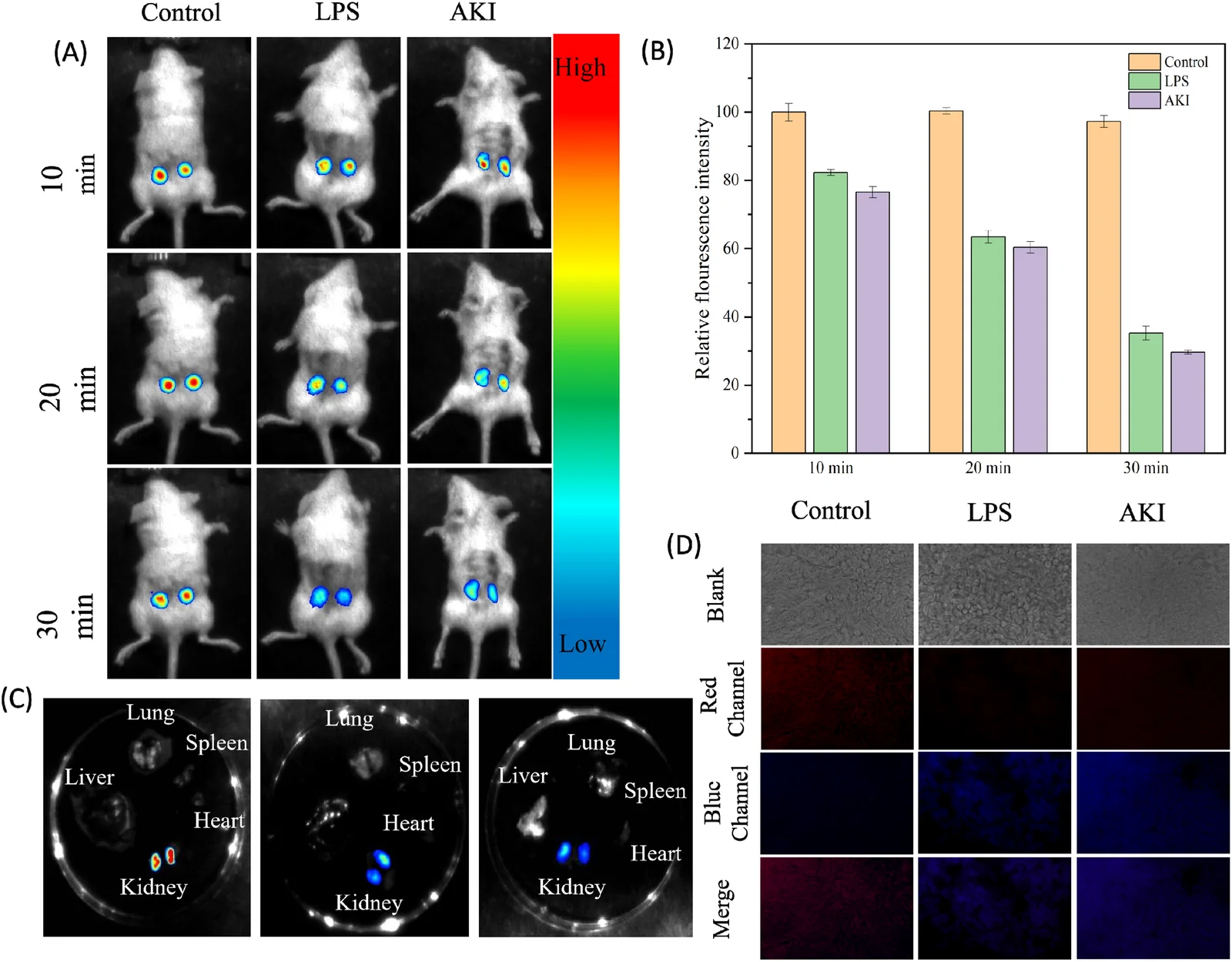
AKI mouse real-time fluorescence imaging. (A) The first column is the imaging of normal mice after intravenous tail injection of nanoprobe; the second column is the imaging of the normal mice after intraperitoneal injection of LPS stimulation and then intravenous injection of nanoprobe; the third column is the imaging of the nanoprobe after intraperitoneal injection of cisplatin to stimulate normal mice and then intravenous injection of nanoprobe. (B) Quantified of relative fluorescence intensity. Error bars: mean ± SD, *n* = 3. Scar bar: 150 µm. *λ*_ex_ = 560 nm. **: *p* < 0.05. (C) Fluorescent imaging of various organs of mice after dissection of three groups: control group, LPS stimulation group and cisplatin stimulation group, respectively, scar bar: 150 µm, *λ*_ex_ = 560 nm. (D) Fluorescence imaging of kidney slices from three groups of mice after dissection: control group, LPS stimulation group and cisplatin stimulation group, respectively. Scale bar: 150 µm, *λ*_ex_ = 380 nm.

Finally, we established an AKI mouse model *via* intraperitoneal administration of cisplatin, utilizing two independent and widely accepted induction paradigms: (1) LPS administration to model sepsis-induced (inflammatory) AKI, and (2) cisplatin administration to model drug-induced (toxic) AKI. These models represent distinct etiologies of AKI—inflammatory and toxic, respectively—and are both well-established and rigorously validated in the literature.^39–42^ The consistent findings observed across both models (Fig. 5) robustly support the conclusion that CHI-CBO reliably detects HClO, a shared pathological hallmark, in diverse AKI subtypes. For *in vivo* fluorescence imaging, we employed an excitation wavelength of 560 nm. Although the probe exhibits maximal absorption at 375 nm, we deliberately selected 560 nm to minimize tissue autofluorescence and light scattering—two major challenges hindering deep-tissue imaging fidelity. While this choice inherently reduces absolute signal intensity, it significantly improves the signal-to-noise ratio, thereby enhancing detection sensitivity and reliability *in vivo*. As shown in Fig. 5A, this strategy enabled clear visualization of a markedly accelerated decay of red fluorescence signal in the renal region of AKI mice compared with healthy controls. We acknowledge that a promising direction for future probe optimization lies in rational structural engineering—specifically, extension of the A–π–A conjugated system—to red-shift the absorption maximum and thereby improve compatibility with standard *in vivo* imaging instrumentation and protocols. In the group, real-time imaging results showed that the CHI-CBO was mainly distributed in the kidney area, and the fluorescence intensity did not decrease significantly within 30 min (Fig. 5: first column), In contrast, in the LPS-stimulated group (Fig. 5A, second column), fluorescence signal intensity progressively decreased over time, with a decrease of ∼40% within 30 min (Fig. 5B), and for the AKI model constructed by cisplatin (Fig. 5: third column), the fluorescence intensity decreased by ∼60% (Fig. 5B). Finally, after dissecting the mice, and taking organs (Fig. 5C) and the kidney tissue (Fig. 5D) of the mice for fluorescence imaging, the same experimental results obtained, the blue channel of the normal group was very weak, and the red channel had a strong red fluorescence signal (Fig. 5C and D), while in the LPS and cisplatin stimulated model, the fluorescence signal in the blue channel was enhanced, and the red signal intensity in the red channel was weakened (Fig. 5C and D). Therefore, the nanoprobe could achieve *in situ* real-time imaging of AKI, and it showed obvious advantages and potential for clinical application in the early diagnosis and monitoring of AKI.

Moreover, it is noteworthy that glycol chitosan—the polymeric scaffold of CHI-CBO—is a well-established, biocompatible, and biodegradable material with a robust safety profile extensively documented in biomedical applications.^43–45^ Its intrinsic mucoadhesive properties and optimal molecular weight collectively facilitate favorable renal accumulation without the need for additional targeting ligands,^46–48^ as clearly demonstrated by the predominant kidney localization observed in Fig. 5C. Furthermore, cytotoxicity assays (Fig. S2) confirm the excellent biocompatibility of CHI-CBO at concentrations significantly exceeding those employed for *in vivo* imaging. While comprehensive biodistribution studies and long-term toxicity assessments would be highly valuable to support future translational development, the present proof-of-concept study successfully establishes the underlying working principle and validates the functionality of the nanoprobe; the existing data already robustly support its safety and sustained renal retention profile.

## 4 Conclusion

In summary, we have successfully developed CHI-CBO—a hypochlorous acid (HClO)-activatable, ratiometric fluorescent nanoprobe. This probe exhibits exceptional selectivity and sensitivity toward HClO, enabling reliable detection in both lipopolysaccharide (LPS)-stimulated cells and live zebrafish. Notably, by capitalizing on the favorable biocompatibility, mucoadhesiveness, and renal accumulation properties of chitosan, CHI-CBO achieves prolonged renal retention and facilitates real-time, quantitative ratiometric imaging of endogenous HClO dynamics in murine models of acute kidney injury (AKI) induced by either LPS or cisplatin. Owing to its robust analytical performance and *in vivo* applicability, CHI-CBO represents a promising molecular tool for elucidating the multifaceted roles of HClO in AKI pathogenesis and related inflammatory and oxidative stress-associated disorders.

We acknowledge that the current *in vivo* imaging strategy relies on monitoring the decay kinetics of the red-channel signal, as the blue-channel emission is undetectable by our IVIS system under the employed excitation wavelength (560 nm). Although this approach is less convenient than instantaneous dual-channel ratiometric imaging, the signal decay rate affords a dynamic readout of pathological hypochlorous acid (HClO) production in the kidney—potentially offering greater pathophysiological insight than a static snapshot for tracking disease progression. Future efforts will focus on developing a dual-channel near-infrared probe to enable real-time ratiometric imaging and streamline the diagnostic workflow. Meanwhile, the current methodology necessitates monitoring signal decay kinetics over a 30 minutes time course, this temporal resolution affords valuable dynamic insights into pathological hypochlorous acid (HClO) production—information that cannot be obtained from single time-point measurements. The high initial signal observed in healthy controls is anticipated and primarily reflects efficient probe delivery; critically, diagnostic information is derived from the decay kinetics rather than from absolute signal intensity. Future efforts will focus on developing dual-channel near-infrared (NIR) probes to enable instantaneous ratiometric imaging, thereby further streamlining the assay procedure.

## Ethical statement

For animal: animal welfare and experimental procedures were carried out in accordance with the guide for the care and use of laboratory animals (Ministry of Science and Technology of China, 2006), and were approved by the animal ethics committee of Central South University. For human: this article does not contain any studies with human participants by any of the authors.

## Author contributions

Tianhui Wu: investigation; methodology; validation; writing – original draft. Zhihui Li: conceptualization; supervision; funding acquisition. Liang Zhang: data curation; formal analysis. Zhijuan Kang: visualization; validation. Mai Xun: visualization; validation. Hanyao Hua: data curation; visualization; validation. Wei Zhang: investigation; methodology. All authors have read and approved the final manuscript.

## Conflicts of interest

Authors have no conflicts of interest.

## Supplementary Material

RA-016-D6RA04230A-s001

## Data Availability

The data supporting this study's findings are available from the corresponding author upon reasonable request. Supplementary information (SI) is available. See DOI: https://doi.org/10.1039/d6ra04230a.
