# Supplementary material for: Construction of an activatable ratiometric fluorescent nanoprobe for HClO detection and imaging in acute kidney injury models
Source: RSC Adv. 2026 Jul 8. Online ahead of print. doi: 10.1039/d6ra04230a (PMC13343533; doi:10.1039/d6ra04230a)
Supplement: RA-OLF-D6RA04230A-s001 [file RA-OLF-D6RA04230A-s001.pdf]

Supplementary Information (SI) for RSC Advances.
This journal is © The Royal Society of Chemistry 2026

Construction of an activatable ratiometric fluorescent nanoprobe for HClO detection and imaging in acute kidney injury models

Tianhui Wu[1], Zhihui Li[1,*], Liang Zhang[1], Zhijuan Kang[1], Mai Xun[1], Hanyao Hua[2], and Wei Zhang[2]

[1]Department of Nephrology, Rheumatology and Immunology, The Affiliated Children's Hospital of Xiangya School of Medicine, Central South University (Hunan children's hospital)，Changsha，Hunan，410007，China

[2]College of Food Science and Engineering, Central South University of Forestry and Technology, Changsha, Hunan 410004, China

Correspondence

Zhihui Li: The Affiliated Children's Hospital of Xiangya School of Medicine, Central South University (Hunan Children's Hospital), Changsha, Hunan, 410007, China. Email: Lizh0731@aliyun.com

1 Experimental Section

1.1 Chemicals and reagents

Glycol chitosan (Mw = 250,000, degree of deacetylation = 82.7%) was purchased from Sigma-Aldrich. Malononitrile, isophorone, sodium thiophenolate, 2-Methyl-2-propanyl bromoacetate, hexamethylenetetramine, CF_3COOH, 1-ethyl-3-(3-(dimethylamino) propyl) carbodiimide hydrochloride (EDC) and N-hydroxysuccinimide (NHS) were purchased from Energy Chemical. All other reagents and solvents are of analytical grade and used without further purification.

1.2 Instruments and measurements

^{1}H NMR spectra were recorded on a Bruker Advance (400 MHz) spectrometer with deuterated chloroform (DMSO-*d6*) as solvents. Transmission electron microscopy (TEM) images were obtained with a JEOL JEM-2100F instrument operated at 200 kV. Dynamic light scattering (DLS) measurements were carried out on Malvern Zetasizer Nano ZS apparatus equipped with a 633 nm He-Ne laser. To determine the hydrodynamic diameter and study the stability of the nanoprobe, samples at different concentration in pH 7.4 PBS and 10% FBS were measured at a scattering angle of 90º at 25 ºC. The sample was diluted to a final concentration of 20 μg/mL in the buffer at pH 7.4. UV-Vis spectra were measured on a Shimadzu UV-2600 spectrometer. All fluorescence measurements were performed using the G9800A fluorescence spectrometer, with both excitation and emission slits fixed at 5 nm, respectively. The fluorescence measurements were taken at an excitation wavelength of 370 nm, and the emission was monitored from 380 to 800 nm.

1.3 Synthetic HClO-activated small molecule fluorescent probe

As depicted in Scheme S1, sodium phenylthiophenolate (2.0 g, 15.2 mmol) and tert-butyl bromoacetate (2.6 mL) were added into absolute DMF (20 mL), which was then reaction at 45 ^{o}C until the starting materials reacted completely (3 h). The solvent of reaction was subsequently evaporated via vacuum, and the residue was purified by PE and DCM as eluent through flash chromatography (PE:DCM = 3:2, v/v). Finally, a white oily product 2 was obtained (2.1 g, 93.8%). The compound structure was not characterized and was directly used for the next step of synthesis.

Compound 1 (1.12 g, 5 mmol) and hexamethylenetetramine (1.4 g, 10 mmol) were added into F_3CCOOH (20 mL), which was then refluxed until the starting materials reacted completely (4 h). The solvent of reaction was subsequently evaporated via vacuum, and the residue was purified by CH_2Cl_2 (DCM) and methyl alcohol as eluent through flash chromatography (DCM:MeOH = 5:1, v/v). Finally, a yellow solid product was obtained (0.85 g, 86.73%). ^{1}H NMR (400 MHz, DMSO-d_6) δ 10.07 (s, 1H), 7.42-7.40 (d, J = 8.01 Hz, 2H), 7.32-7.28 (t, J=8.02 2H), 7.25-7.21 (t, J=8.00, 1H), 3.66 (s, 2H), ^{13}C NMR (100 MHz, DMSO-d_6) δ 213.48, 175.61, 134.370, 129.97, 129.06, 127.41, 42.12.

2-(3,5,5-trimethylcyclohex-2-en-1-ylidene) malononitrile (0.5 g, 2.7 mmol), compound **2** (0.53 mg, 2.7 mmol) and 5 drops of piperidine were added into absolute CH_3CH_2OH (20 mL), which was then refluxed until the starting materials reacted completely (3.5 h). The solvent of reaction was subsequently evaporated via vacuum, and the residue was purified by DCM and methyl alcohol as eluent through flash chromatography (DCM:MeOH = 2:1, v/v). Finally, an orange solid product was

obtained (0.62 g, 65.6%). ^{1}H NMR (400 MHz, DMSO-*d6*) δ 7.43-7.41 (d, *J* = 8.01 Hz, 2H), 7.26-7.22 (t, *J* = 4.00Hz, 2H), 7.03-6.92 (dd, J=16.01, 2H), 6.82 (s, 1H), 3.58 (s, 2H), 2.59 (s, *2*H), 2.46 (s, 2H), 1.08 (s, 6H). ^{13}C NMR (100 MHz, DMSO-*d6*) δ 191.36, 169.26, 153.81, 141.30, 136.36, 132.14, 130.14, 128.24, 127.93, 126.14, 125.21, 123.21, 113.62, 112.69, 42.97, 39.18, 31.81, 28.02, 15.29.

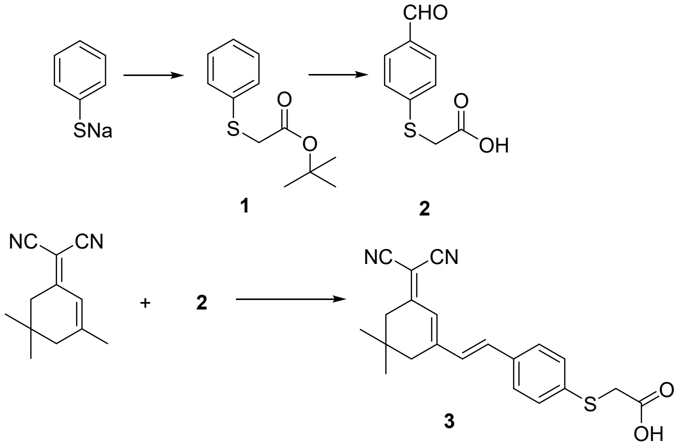

Scheme S1. Synthetic route map of small molecule probe **3**.

1.4 Synthetic HClO-activated nano fluorescent probe

To obtain CHI-CBO, glycol chitosan (2.5 g, 0.10 mmol) was completely dissolved in 25 mL of deionized water, and then the DMSO solution containing **3** (36.45 mg, 0.10 mmol), EDCI (23.00 mg, 0.12 mmol), and NHS (13.81 mg, 0.12 mmol) was added. The resulting solution was purified by dialysis in 1:4 (v/v) DMSO/H2O for 1 day, and CHI-CBO was allowed to self-assemble into nanoscale micelles by further dialysis in deionized water for 1 day (MWCO: 14 kDa). The resulting nanoparticles were dispersed by freeze-drying or used directly for characterization. The dried CHI-CBO nanoprobes could be re-dispersed in water at a concentration of 10 mg/mL after a few minutes of ultrasonic treatment for further use.

1.5 Cell culture and in vitro cell toxicity assay

RAW264.7 cells were cultured in Dulbe's modified Eagle's medium (DMEM) with 10% fetal bovine serum (FBS), penicillin (100 U/ml) and streptomycin (100 μg/mL) at 37ºC in a humidified atmosphere of CO_2/air (5:95). For cytotoxicity, RAW264.7 cells were seeded in 96-well plates at an initial density of 5000 cells/well in 120 μL of complete DMEM was added to each well. After incubation for 24 h at 37ºC, the DMEM was replaced with fresh medium, and cells were treated with nanoprobe CHI-CBO at different concentrations and incubated at 37ºC for 24 h. Then, 20 μL of MTT (3-(4,5-Dimethylthiazol-2-yl)-2,5-diphenyltetrazolium bromide 5 mg/mL in PBS) was added to each well. After incubation for 4 h, the medium was removed, and 100 μL of DMSO was added to dissolve the blue formazan crystals, then gently shaken for 15 min. The absorbance was recorded at 490 nm using a microplate reader (Bio-Tek).

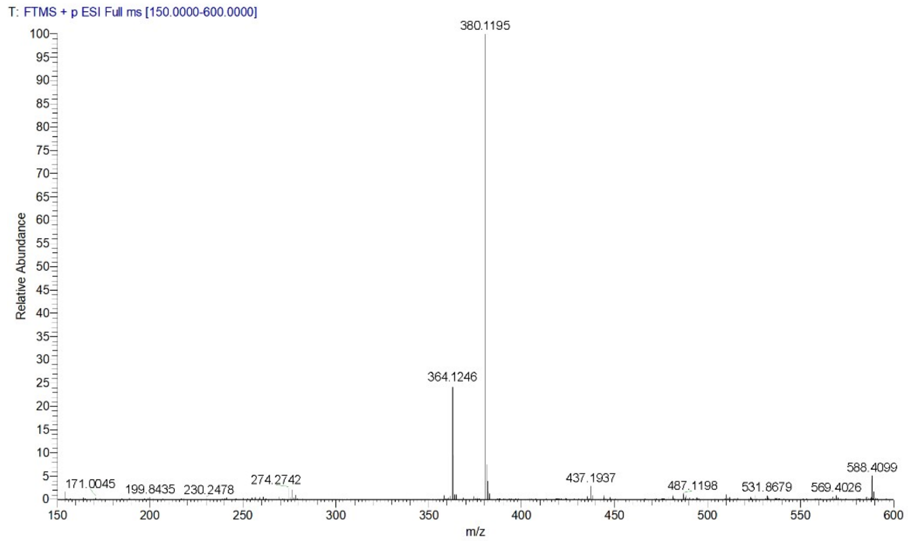

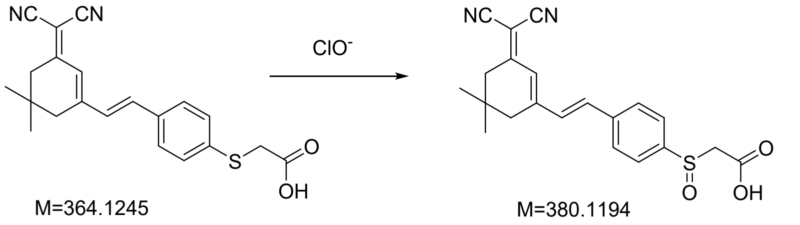

Figure S1. Demonstrate the response mechanism of small molecule probe with ClO^- using mass spectrometry. The calculated m/z values for $[M+H]^+$ are 364.1245 and 380.1194, respectively.

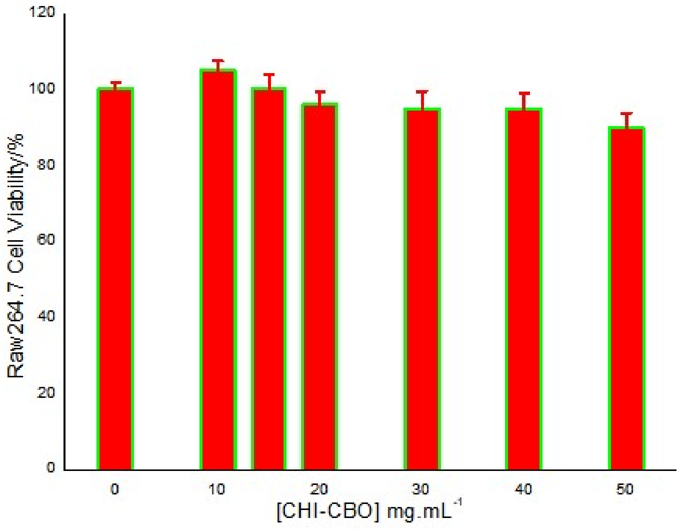

Figure S2. RAW264.7 cell toxicity of different concentration (0-50) mg/mL of nanoprobe CHI-CBO.

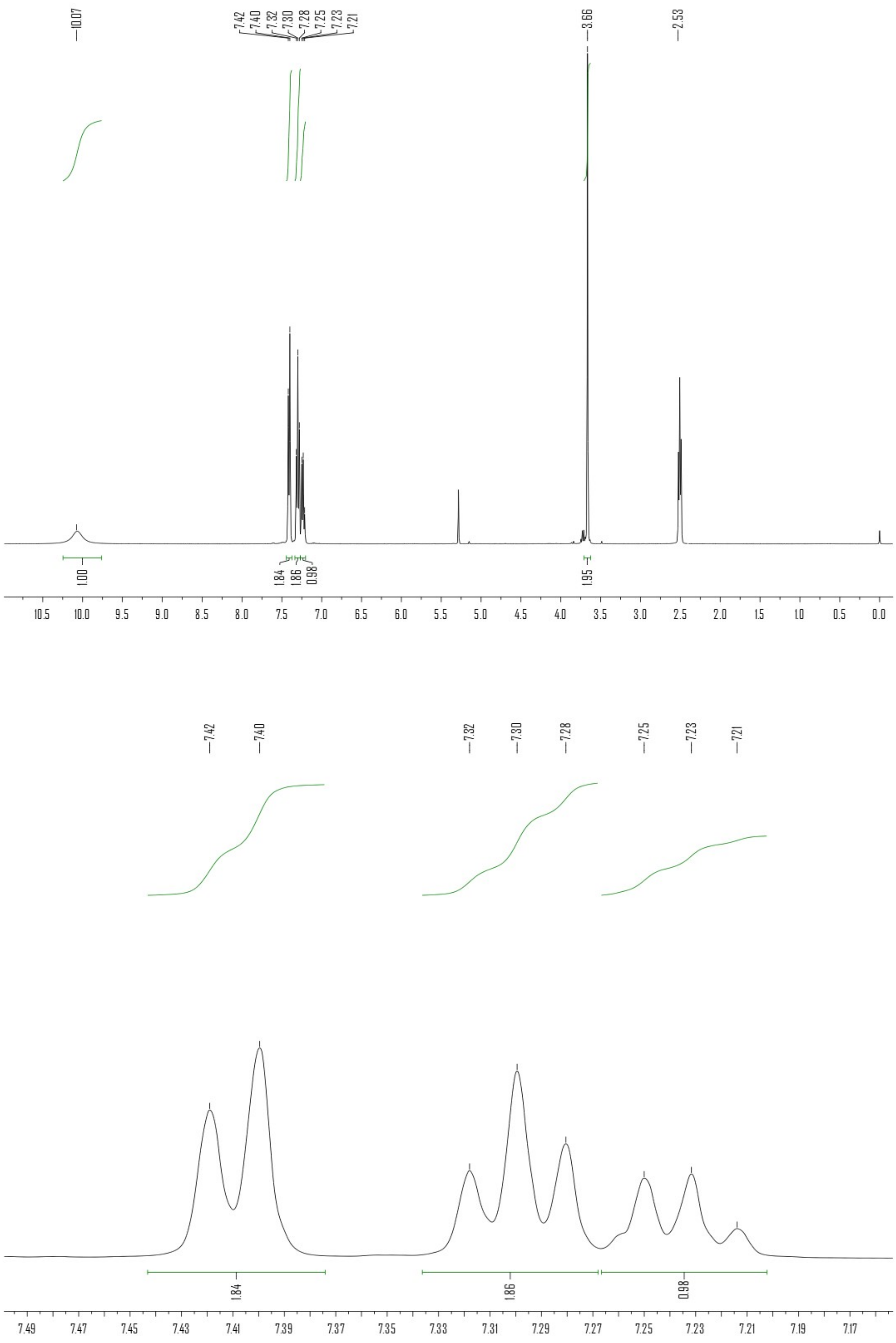

Figure S3. ^{1}H NMR of 2.

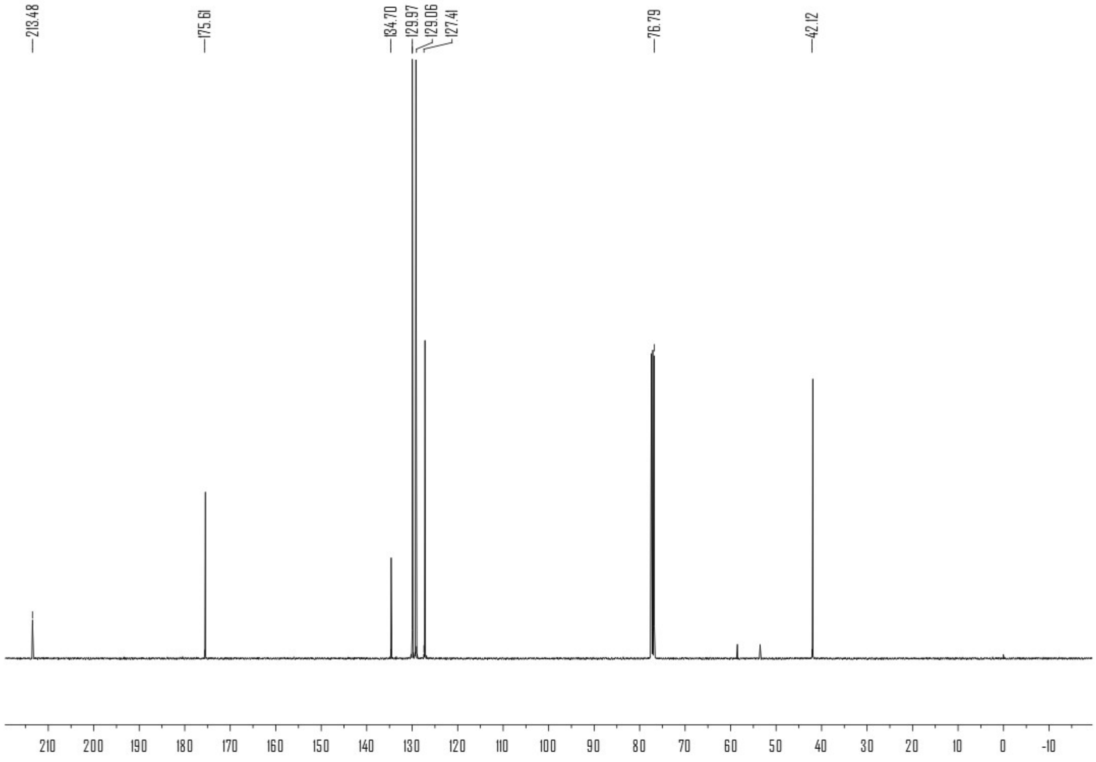

Figure S4. ^{13}C NMR of 2

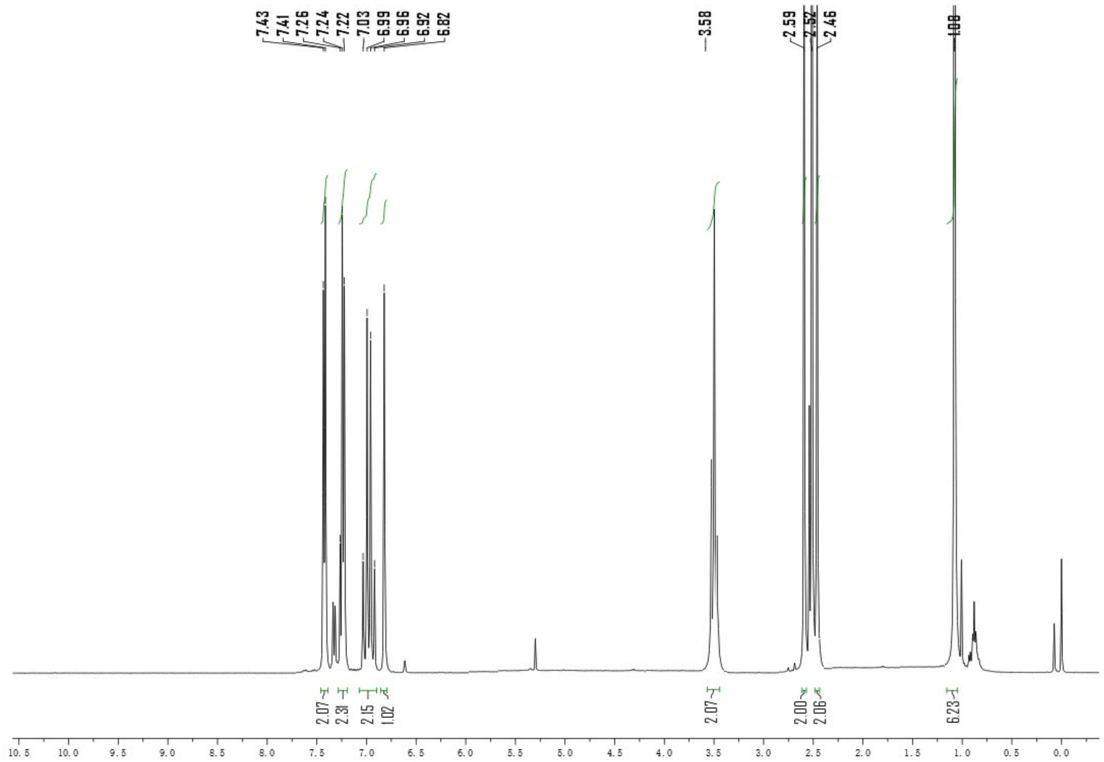

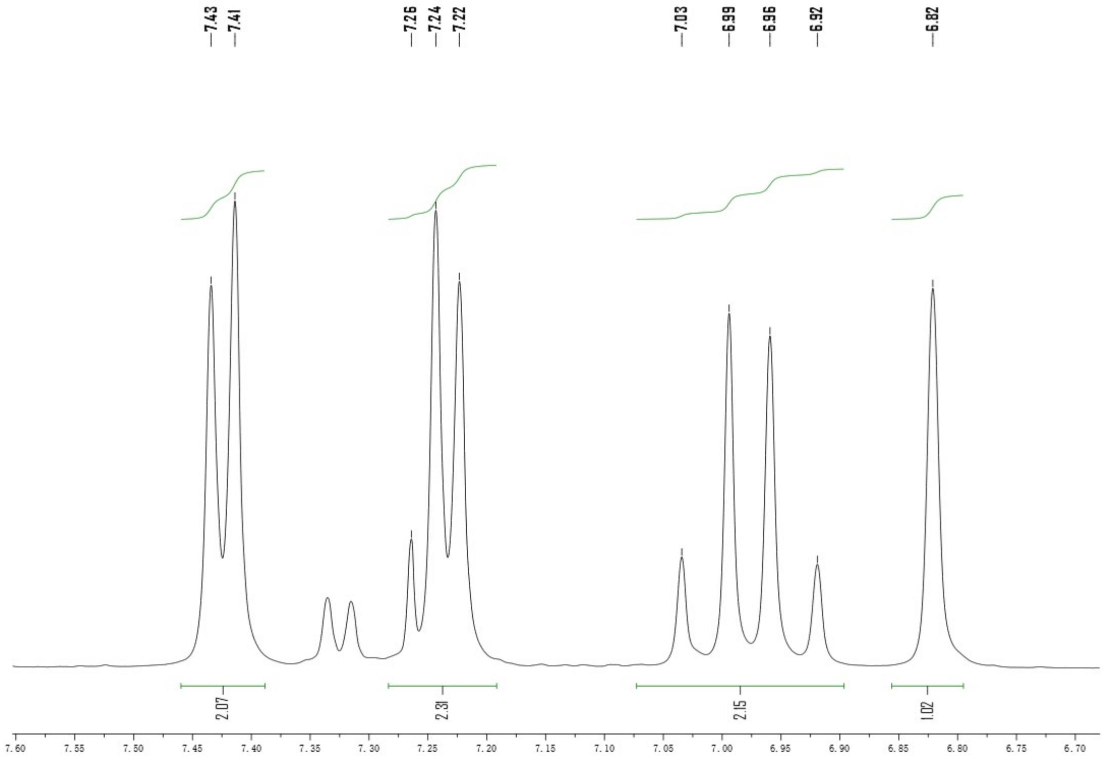

Figure S5. ^{1}H NMR of 3

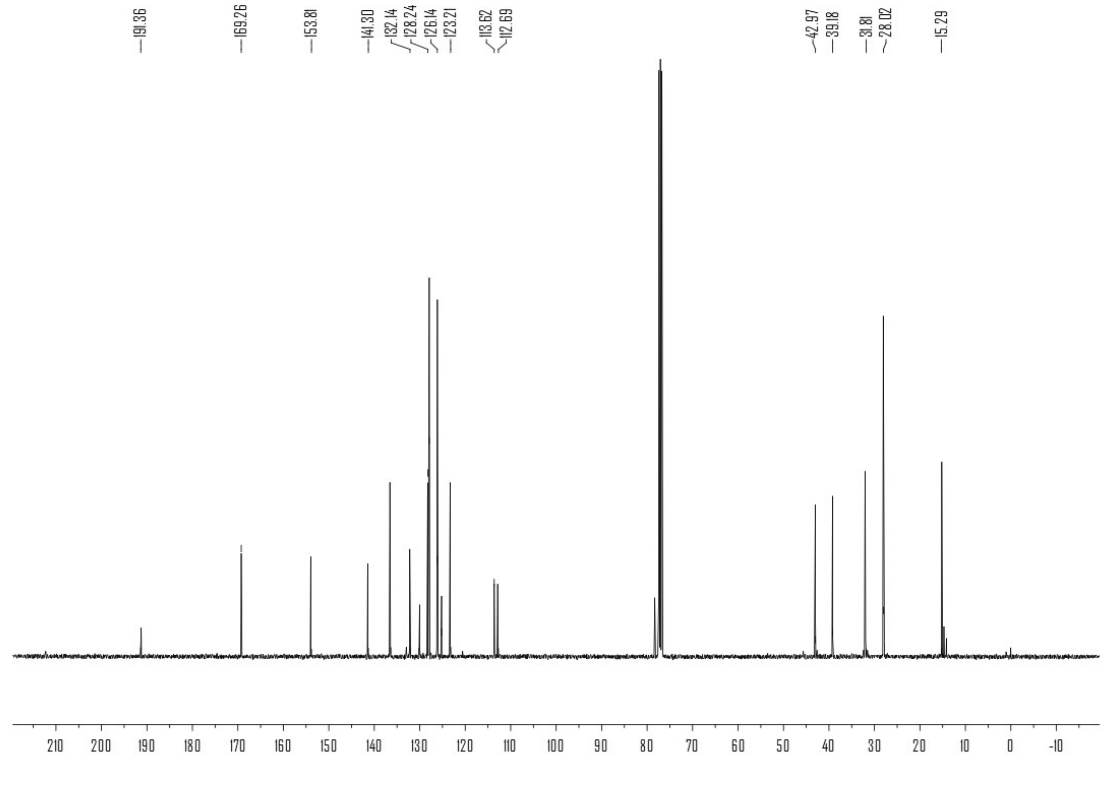

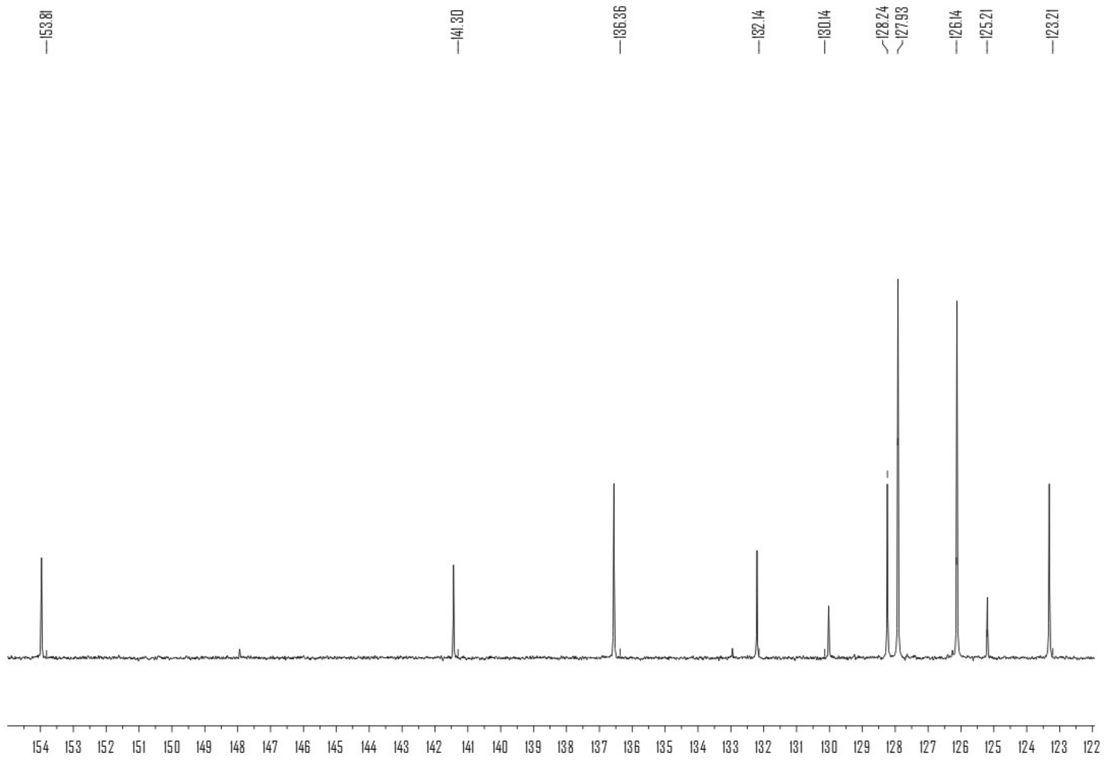

Figure S6. ^{13}C NMR of 3
